# The Value of a Cystatin C-based Estimated Glomerular Filtration Rate for Cardiovascular Assessment in a General Japanese Population: Results From the Iwate Tohoku Medical Megabank Project

**DOI:** 10.2188/jea.JE20180274

**Published:** 2020-06-05

**Authors:** Takuya Osaki, Mamoru Satoh, Fumitaka Tanaka, Kozo Tanno, Yuji Takahashi, Takahito Nasu, Kiyomi Sakata, Yoshihiro Morino, Kenji Sobue, Makoto Sasaki

**Affiliations:** 1 Division of Cardiology, Department of Internal Medicine, Iwate Medical University, Iwate, Japan; 2 Division of Biomedical Information Analysis, Institute for Biomedical Sciences, Iwate Medical University, Iwate, Japan; 3 Division of Biobank and Data Management, Iwate Tohoku Medical Megabank Organization, Iwate Medical University, Iwate, Japan; 4 Division of Clinical Research and Epidemiology, Iwate Tohoku Medical Megabank Organization, Iwate Medical University, Iwate, Japan; 5 Division of Biomedical Information Analysis, Iwate Tohoku Medical Megabank Organization, Iwate Medical University, Iwate, Japan; 6 Division of Nephrology and Hypertension, Department of Internal Medicine, Iwate Medical University, Iwate, Japan; 7 Department of Hygiene and Preventive Medicine, Iwate Medical University, Iwate, Japan; 8 Deputy Executive Director, Iwate Tohoku Medical Megabank Organization, Disaster Reconstruction Center, Iwate Medical University, Iwate, Japan; 9 Executive Director, Iwate Tohoku Medical Megabank Organization, Disaster Reconstruction Center, Iwate Medical University, Iwate, Japan; 10 Department of Neuroscience, Institute for Biomedical Sciences, Iwate Medical University, Iwate, Japan; 11 Division of Ultrahigh Field MRI, Institute for Biomedical Sciences, Iwate Medical University, Iwate, Japan

**Keywords:** chronic kidney disease, high-sensitivity cardiac troponin T, N-terminal pro-brain natriuretic peptide, urine albumin-to-creatinine ratio, Suita score

## Abstract

**Background:**

Epidemiological studies have shown that high circulating cystatin C is associated with a risk of cardiovascular disease (CVD) independent of creatinine-based renal function measurements. The present study investigated the comparison between the cystatin C-based estimated glomerular filtration rate (GFRcys) and creatinine-based GFR (GFRcr) to determine whether these measurements are associated with CV biomarkers and elevated CVD risk in a general Japanese population.

**Methods:**

The Iwate Tohoku Medical Megabank Organization pooled individual participant data from a general population-based cohort study in Iwate prefecture (*n* = 29,375). Chronic kidney disease (CKD) was estimated using the GFRcys, GFRcr and the urine albumin-to-creatinine ratio (UACR).

**Results:**

The prevalence of CKD in the participants was found to be higher based on the GFRcr than the GFRcys. Multiple variable analyses after adjusting for baseline characteristics showed that high-sensitivity cardiac troponin T (hs-cTnT) and N-terminal pro-brain natriuretic peptide (NT-proBNP) were associated with the GFRcys. The area under the receiver operating characteristic (AUROC) curve for identifying individuals with a high Suita score was higher for the GFRcys (AUROC = 0.68) than it was for the GFRcr (AUROC = 0.64, *P* < 0.001). The GFRcys provided reclassification improvement for the CVD risk prediction model by the GFRcr (net reclassification improvement = 0.341; integrated discrimination improvement = 0.018, respectively, *P* < 0.001).

**Conclusions:**

The GFRcys is more closely associated with CV biomarkers, including hs-cTnT and NT-proBNP levels, and a high Suita score than the GFRcr, and it provides additional value in the assessment of CVD risk using GFRcr.

## INTRODUCTION

Chronic kidney disease (CKD) has been defined as abnormalities in kidney structure or function and/or a glomerular filtration rate (GFR) of less than 60 mL/min/1.73 m^2^ and/or a urine albumin-to-creatinine ratio (UACR) over 30 mg/g Cr for more than 3 months by the Kidney Disease Improving Global Outcomes (KDIGO) CKD guideline 2012.^1^ It has been established that the presence of renal dysfunction is related to the onset of cardiovascular disease (CVD) and mortality.^2^^–^^4^

It is well known that GFR is measured using two estimates. The first estimate is based on the serum creatinine level, while the second estimate is derived from serum cystatin C levels.^5^ It has been reported that the cystatin C-based renal function measurement is more accurate than the creatinine-based measurement in patients with mild or moderate CKD.^6^ However, it is unclear whether there is a difference between cystatin C- and creatinine-based renal function measurements in a general Japanese population. It is also unclear whether the cystatin C-based estimated GFR (GFRcys) and/or creatinine-based estimated GFR (GFRcr) are related to cardiovascular (CV) biomarkers in a general Japanese population.

On March 11, 2011, the Great East Japan Earthquake and resulting tsunami caused devastating damage to the Pacific coast of the Tohoku region. The Tohoku Medical Megabank Project (TMM), which was conducted by Iwate Medical University and Tohoku University, was launched to implement creative reconstruction and to solve medical problems during the aftermath of this disaster.^7^ The TMM started a population-based adult cohort study, the TMM Community-Based Cohort Study, which recruited participants from a general Japanese population.^7^ The present study investigated differences in renal function measurements between the GFRcys and GFRcr and sought to determine whether the GFRcys and/or GFRcr are related to CV biomarkers and CVD risk in the Iwate TMM Community-Based Cohort Study.

## METHODS

### Study population

The TMM initiated a population-based adult cohort study named the TMM Community-Based Cohort Study in May 2013.^7^ The TMM participants were recruited from Iwate and Miyagi prefectures from May 2013 to March 2016.^7^ The population of the present study included individuals from 20 to 74 years of age living in Iwate. Participants were excluded from the study if they had self-reported kidney disease, were undergoing a kidney transplant, were pregnant, or were infected with human immunodeficiency virus (HIV), an autoimmune disorder or a malignant or primary wasting disorder. The present study was approved by the ethics committee of the Iwate Medical University School of Medicine, and written informed consent was obtained from all participants.

### Cohort data collection

All participants completed self-administered questionnaires and underwent standardized interviews conducted by trained research staff who collected information concerning medical history and medication. Blood samples and a random spot urine sample were collected. Histories of stroke, coronary artery disease (CAD), and heart failure (HF) were defined through the self-reported history. Hypertension was defined as a systolic blood pressure (BP) ≥140 mm Hg, diastolic BP ≥90 mm Hg, being diagnosed with hypertension, and/or the use of antihypertensive medication.^8^ Diabetes was defined as glycated hemoglobin (HbA1c) value ≥6.5%, a nonfasting glucose concentration ≥200 mg/dL, being diagnosed with diabetes, and/or undergoing treatment with antidiabetic drugs, including insulin.^9^ Dyslipidemia was defined as low-density lipoprotein (LDL) cholesterol ≥140 mg/dL, being diagnosed with dyslipidemia, and/or the use of antihyperlipidemic medication.^10^ Hyperuricemia was defined as uric acid ≥7.0 mg/dL, being diagnosed with hyperuricemia, and/or undergoing treatment with antihyperuricemics.^11^

### Measurements of CV biomarkers

Peripheral blood samples were collected from the upper arm while participants were in a seated position after resting. Serum samples were obtained from participants and used to measure cystatin C, high-sensitivity cardiac troponin T (hs-cTnT), and N-terminal pro-brain natriuretic peptide (NT-proBNP) levels. Serum cystatin C levels were measured using an assay with a calibration traceable to the international standard reference material (Auto cystatin C; BML, Saitama, Japan). Serum levels of hs-cTnT were measured using an EcLusys high-sensitivity troponin T assay (Roche Diagnostics K.K., Tokyo, Japan). The lower detection limits were 0.40 mg/L for the cystatin C assay and 3 pg/mL for the hs-cTnT assay, both of which were used as the cutoff points in the present analysis. The 99^th^ percentile value of hs-cTnT in an apparently healthy population has been reported to be 14 pg/mL. In addition, serum levels of NT-proBNP were determined using the EcLusys NT-proBNP II assay (Roche Diagnostics K.K., Tokyo, Japan). The lower detection limit of the NT-proBNP assay was 125 pg/mL. Urine albumin concentrations were corrected to urine creatinine (Cr) concentrations and are expressed as the urine albumin-to-creatinine ratio (UACR). Participants were divided into three stages according to the UACR as follows: A1 = UACR < 30 mg/gCr; A2 = 30–299 mg/gCr; and A3 = UACR ≥ 300 mg/gCr.

### Measurement of GFR

The Japanese equation for the GFR is as follows: GFRcr = 194 × SCr^−1.094^ × Age^−0.287^ [× 0.739 if female] (mL/min/1.73 m^2^), GFRcys = 104 × ScysC^−1.019^ × 0.996^Age^ [× 0.929 if female] − 8 (mL/min/1.73 m^2^).^12^^,^^13^ We applied Tanaka’s equation to calculate the estimated amount of salt intake using a spot urine sample.^14^ The equation is as follows: {21.98 × Urine Na (mEq/L)/Urine Cr (mg/dl) × 1/10 × [Body weight (kg) × 14.89 + Height (cm) × 16.14 − Age × 2.04 − 2244.45]}^0.392^ × 1/17 (g/day).^14^ CKD was defined as a GFR less 60 mL/min/1.73 m^2^ and/or a UACR over 30 mg/gCr.^1^ CKD was classified into the following five stages according to the GFR: G1 = GFR ≥ 90 mL/min/1.73 m^2^; G2 = GFR 60–89 mL/min/1.73 m^2^; G3a = GFR 45–59 mL/min/1.73 m^2^; G3b = GFR 30–44 mL/min/1.73 m^2^; G4 = GFR 15–29 mL/min/1.73 m^2^; G5 = GFR < 15 mL/min/1.73 m^2^.^1^

### Definition of CVD risk

The present study performed the evaluation of CVD risk using the Suita score because it has been shown that the Suita score was more accurate for predicting coronary heart disease (CHD) in the Japanese population than the Framingham risk score.^15^ The Suita score (LDL Suita score) for subjects ≥35 years of age (*n* = 28,288) was calculated according to the formula accounting for age, sex, current smoking, diabetes, BP, LDL-cholesterol, high-density lipoprotein (HDL)-cholesterol, and CKD stage (defined using GFRcr).^15^ The subjects who showed a Suita score (over 56) for a 9% or higher predicted probability of CHD in 10 years were defined as high-risk subjects for CVD.^10^

### Statistical analysis

Continuous variables are expressed as the means (standard deviations [SDs]) or medians (25^th^–75^th^ percentiles). Bland-Altman plots displayed the difference between GFRcr and GFRcys for each person. Pearson’s correlation coefficient was used to assess the correlation between GFRcr and GFRcys. Differences in the baseline levels of the clinical characteristics at different CKD stages based on the GFRcr and GFRcys were analyzed using one-way analysis of variance (one-way ANOVA) with Tukey’s or the Games-Howell post hoc tests, or the Kruskal-Wallis test as appropriate. The *t*-test and chi-squared test were also performed for categorical variables. Multiple regression models were used to analyze the association between CVD biomarkers and values of the GFRcys or GFRcr. The serum levels of NT-proBNP, hs-cTnT, and UACR were logarithmically transformed for statistical analyses. Analyses of the receiver operating characteristic (ROC) curves were performed to examine the sensitivity and specificity of the values of each GFR for detecting high-risk subjects for CVD. The area under the receiver operating characteristic (AUROC) curves were compared using the DeLong test, net reclassification improvement (NRI), and integrated discrimination improvement (IDI). Analyses were performed using statistical software (SPSS 23; IBM Corp, Armonk, NY, USA), Excel 2013 and R (ver. 3.4.4; R Foundation for Statistical Computing, Vienna, Austria) for Windows. A two-sided value of *P* < 0.05 was used to indicate significance.

## RESULTS

### Baseline characteristics

Baseline characteristics are shown in Table 1. The Iwate TMM Community-Based Cohort Study recruited 32,675 participants from the Iwate prefecture and excluded 3,300 participants due to past history that included dialysis, CAD, or stroke, or lack of some baseline characteristics or laboratory data. Ultimately, the present study included 29,375 participants.

**Table 1.** Baseline characteristics of the participants

Parameter	Value
Number of participants	29,375
Age, years^a^	59.8 (11.4)
Male, %	35.7
BMI, kg/m^2 a^	23.5 (3.6)
Hypertension, %	41.2
Diabetes, %	8.8
Dyslipidemia, %	32.1
Hyperuricemia, %	8.8
Current smoker, %	24.9
HF, %	0.2
Cancer, %	7.2
Serum creatinine, mg/dL^a^	0.66 (0.17)
Serum cystatin C, mg/L^a^	0.71 (0.14)
UACR, mg/gCr^b^	7.7 (4.9–14.7)
Estimated salt intake amount, g/day^a^	10.1 (2.3)
Systolic blood pressure, mm Hg^a^	127.2 (18.1)
NT-proBNP, pg/mL^b^	48.0 (28.0–81.0)
Hs-cTnT, pg/mL^b^	5.0 (3.0–8.0)
HbA1c, %^a^	5.7 (0.6)
Triglycerides, mg/dL^a^	126.2 (81.1)
HDL-cholesterol, mg/dL^a^	64.3 (16.9)
LDL-cholesterol, mg/dL^a^	120.0 (30.0)
Uric acid, mg/dL^a^	4.9 (1.3)
Suita score (*n* = 28,288, age ≥35 years)^b^	43 (34–50)

BMI, body mass index; HbA1c, glycated hemoglobin; HDL, high-density lipoprotein; HF, heart failure; Hs-cTnT, high-sensitivity cardiac troponin T; LDL, low-density lipoprotein; NT-proBNP, N-terminal pro-brain natriuretic peptide; UACR, urine albumin-to-creatinine ratio.

^a^Mean (standard deviation).

^b^Median (interquartile range).

### Distributions of GFRcys and GFRcr

**Figure 1.**
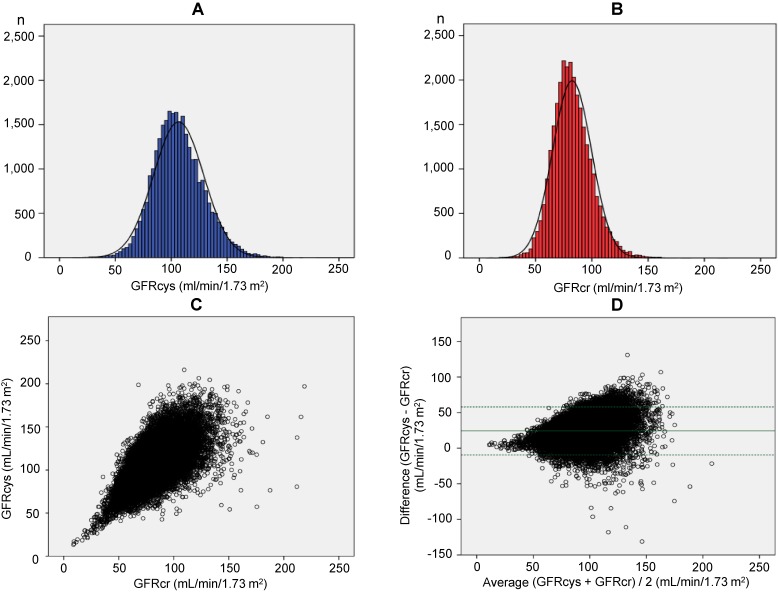
Distributions of the GFRcys (**A**) and the GFRcr (**B**). The mean value of the GFRcys was higher than that of the GFRcr [106.6 (22.5) mL/min/1.73 m^2^ vs 82.4 (17.3) mL/min/1.73 m^2^, GFRcys vs GFRcr, respectively, *P* < 0.001]. The correlation plot between the GFRcys and GFRcr (**C**). There was a positive correlation between the GFRcys and GFRcr (*r* = 0.65, *P* < 0.001). Bland-Altman plots show the difference in GFR between cystatin C-based and creatinine-based equations (**D**). The upper and lower broken lines indicate the reference lines. The middle line indicates the regression line. The marked field signifies the limits of agreement (1.96 SD). GFRcys, cystatin C-based estimated glomerular filtration rate; GRFcr, creatinine-based estimated glomerular filtration rate.

The mean value of the GFRcys was higher than that of the GFRcr in the participants (106.6 [SD, 22.5] mL/min/1.73 m^2^ vs 82.4 [SD, 17.3] mL/min/1.73 m^2^, GFRcys vs GFRcr, respectively, *P* < 0.001] (Figure 1A and Figure 1B). There was a positive correlation between the GFRcys and GFRcr (*r* = 0.65, *P* < 0.001) (Figure 1C). Bland-Altman plots showed the difference in the GFR between cystatin C- and creatinine-based measurements (Figure 1D). There was a large positive or negative difference over one hundred for a high mean GFR between the two equations (Figure 1D).

### Comparison of CKD stages based on GFRcys and GFRcr

Some of these baseline characteristics in CKD stages G2 and G3–5 based on both the GFRcys and GFRcr were higher than those in CKD stage G1 based on both the GFRcys and GFRcr (All *P* < 0.01, Table 2). The prevalence rates of hypertension and hyperuricemia in CKD stage G2 and G3–5 based on both the GFRcys and GFRcr were higher than those in CKD stage G1 (All *P* < 0.01, Table 2). In addition, CV biomarkers, including levels of hs-cTnT, NT-proBNP, and serum uric acid, in CKD stages G2 and G3–5 based on both the GFRcys and GFRcr were higher compared to those in CKD stage G1 (All *P* < 0.01, Table 2).

**Table 2.** Baseline characteristics of each CKD stage based on the GFRcr and the GFRcys

Parameters	GFRcys			GFRcr		
	
	G1	G2	G3–5	G1	G2	G3–5
*N*	22,699	6,282	394	8,699	18,480	2,196
Age, years^a^	57.6 (11.7)	67.3 (5.7)^*^	68.5 (5.6)^#^	53.7 (13.2)	61.8 (9.5)^*^	67.4 (5.8)^#^
Male, %	34.3	40.1^*^	49.2^#^	33.3	36.1^*^	42.1^#^
BMI, kg/m^2 a^	23.3 (3.5)	24.3 (3.6)^*^	25.1 (4.2)^#^	23.2 (3.8)	23.6 (3.5)^*^	24.4 (3.5)^#^
Hypertension, %	36.6	55.8^*^	69.8^#^	33.2	42.7^*^	60.2^#^
Diabetes, %	7.9	11.4^*^	19.3^#^	9.1	8.3	12.2^#^
Dyslipidemia, %	31.6	33.8^*^	29.7	26.4	34.3^*^	35.7
Current smoker, %	24.4	26.4^*^	32.2^#^	21.8	25.9^*^	29.0^#^
Hyperuricemia, %	6.8	14.3^*^	38.8^#^	5.1	9.0^*^	22.6^#^
HF, %	0.2	0.5^*^	1.0	0.1	0.3^*^	0.6
Cancer, %	6.4	9.9^*^	15.5^#^	5.8	7.5^*^	10.9^#^
Creatinine, mg/dL^a^	0.63 (0.13)	0.75 (0.16)^*^	1.17 (0.56)^#^	0.54 (0.09)	0.68 (0.11)^*^	0.97 (0.29)^#^
Cystatin C, mg/L^a^	0.66 (0.08)	0.87 (0.08)^*^	1.35 (0.34)^#^	0.63 (0.09)	0.72 (0.11)^*^	0.95 (0.25)^#^
UACR, mg/gCr^b^	7.4 (4.8–13.3)	8.9 (5.4–19.2)^*^	26.6 (8.7–155.9)^#^	7.8 (5.0–14.4)	7.5 (4.8–14.1)	9.2 (5.2–25.9)^#^
Estimated salt intake amount, g/day^a^	10.2 (2.3)	9.7 (2.3)^*^	9.1 (2.5)^#^	10.5 (2.3)	10.0 (2.2)^*^	9.3 (2.3)^#^
Systolic blood pressure, mm Hg^a^	126.1 (17.9)	130.7 (17.9)^*^	130.6 (19.5)	125.0 (18.3)	127.9 (17.8)^*^	130.1 (18.4)^#^
NT-proBNP, pg/mL^b^	43.0 (25.0–71.0)	67.0 (41.0–112.0)^*^	125.5 (76.0–222.8)^#^	40.0 (24.0–65.0)	50.0 (29.0–83.0)^*^	72.0 (42.0–127.0)^#^
Hs-cTnT, pg/mL^b^	5.0 (1.5–7.0)	7.0 (5.0–10.0)^*^	11.0 (8.0–16.0)^#^	4.0 (1.5–6.0)	5.0 (3.0–8.0)^*^	8.0 (5.0–11.0)^#^
HbA1c, %^a^	5.65 (0.61)	5.78 (0.58)^*^	5.94 (0.84)^#^	5.65 (0.75)	5.69 (0.54)^*^	5.79 (0.55)^#^
LDL-C, mg/dL^a^	120.1 (30.1)	120.3 (29.6)	114.5 (32.5)^#^	116.5 (30.3)	121.8 (29.9)^*^	119.9 (29.3)^#^
Uric acid, mg/dL^a^	4.79 (1.25)	5.40 (1.28)^*^	6.24 (1.40)^#^	4.58 (1.23)	5.00 (1.26)^*^	5.81 (1.31)^#^
Suita score (*n* = 28,288, age ≥35 years)^b^	40 (31–48)	48 (42–54)^*^	52 (46–59)^#^	38 (30–46)	43 (36–50)^*^	51 (44–57)^#^

BMI, body mass index; HbA1c, glycated hemoglobin; HF, heart failure; Hs-cTnT, high-sensitivity cardiac troponin T; LDL, low-density lipoprotein; NT-proBNP, N-terminal pro-brain natriuretic peptide; UACR, urine albumin-to-creatinine ratio.

^a^Mean (standard deviation).

^b^Median (interquartile range).

^*^P < 0.01 vs G1; ^#^*P* < 0.01 vs G2.

### Association between CV biomarkers and GFR

Multiple regression models after adjustment for baseline characteristics and the log of the UACR revealed that increases in CV biomarkers, including uric acid, hs-cTnT, and NT-proBNP, were statistically associated with both the GFRcys and GFRcr, although hs-cTnT was weakly associated with the GFRcr (all *P* < 0.001, Table 3).

**Table 3.** Associations between the CV parameters and the GFR in multiple regression models

Variable	Model 1			Model 2		
	
	95% CI	Beta	*P*	95% CI	Beta	*P*
**GFRcys**						
Uric acid, mg/dL	−3.47 (−3.65 to −3.30)	−0.20	<0.001	−3.45 (−3.63 to −3.27)	−0.20	<0.001
Log hs-cTnT	−3.89 (−4.18 to −3.60)	−0.13	<0.001	−3.78 (−4.06 to −3.49)	−0.13	<0.001
Log NT-proBNP	−3.74 (−3.98 to −3.50)	−0.15	<0.001	−3.60 (−3.84 to −3.36)	−0.14	<0.001
**GFRcr**						
Uric acid, mg/dL	−3.05 (−3.22 to −2.89)	−0.23	<0.001	−3.04 (−3.21 to −2.87)	−0.28	<0.001
Log hs-cTnT	−0.76 (−1.02 to −0.49)	−0.03	<0.001	−0.70 (−0.96 to −0.43)	−0.03	<0.001
Log NT-proBNP	−2.07 (−2.30 to −1.85)	−0.10	<0.001	−1.20 (−2.22 to −1.78)	−0.10	<0.001

Beta, standardized partial regression coefficient; CI, confidence interval; CV, cardiovascular; GFR, glomerular filtration rate; Log hs-cTnT, high-sensitivity cardiac troponin T was logarithmically transformed; Log NT-proBNP, N-terminal pro-brain natriuretic peptide was logarithmically transformed; log UACR, urine albumin-to-creatinine ratio was logarithmically transformed.

Model 1: Adjusted for age, systolic blood pressure, body-mass index, low-density lipoprotein cholesterol, HbA1c, current smoker, hypertension, dyslipidemia, diabetes, hyperuricemia and heart failure.

Model 2: Adjusted for model 1 plus log UACR.

### Comparison of the AUROC curves of the GFR for the discrimination of high-risk subjects for CVD and the ability to predict the CVD risk

**Figure 2.**
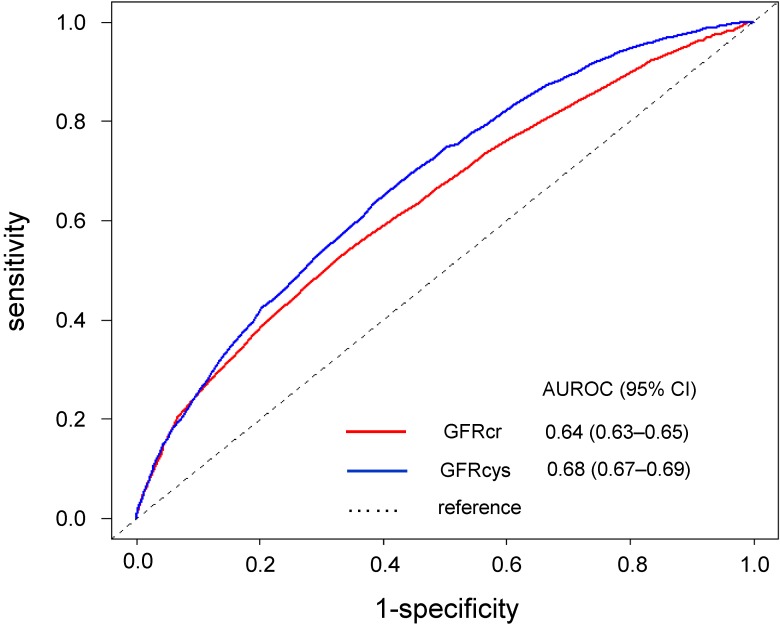
Comparison of AUROC curves of the GFR for the discrimination of high-risk subjects for CVD and the ability to predict the CVD risk. In the evaluation of CVD risk using the Suita score, the subjects who showed a Suita score for a 9% or higher 10-year CHD risk were defined as high-risk subjects for CVD. AUROC, area under the receiver operator curve; CHD, coronary heart disease; CVD, cardiovascular disease; CI, confidence interval; GFRcys, cystatin C-based estimated glomerular filtration rate; GRFcr, creatinine-based estimated glomerular filtration rate.

The AUROC curve for the individuals with a high Suita score (≥56) for the GFRcys (AUROC = 0.68, *P* < 0.001) was larger than that for the GFRcr (AUROC = 0.64, *P* < 0.001, Figure 2). In addition, the GFRcys provided the reclassification improvement for the CVD risk prediction model by the GFRcr (NRI = 0.341, 95% CI, 0.305–0.377; *P* < 0.001; IDI = 0.018, 95% CI, 0.016–0.020; *P* < 0.001, Table 4).

**Table 4.** Reclassification and discrimination improvement for predictors of high risk subjects for CVD by adding the GFRcys to the GFRcr

	Updated Model^**^				% reclassified

	Q1 <7.4%	Q2 7.4≤ to <10.6%	Q3 10.6≤ to <14.1%	Q4 ≥14.1%	
Initial Model^*^					
**Suita Low or Moderate score**					
Q1 <7.4%	4,602	1,070	362	89	25
Q2 7.4≤ to <10.6%	2,881	2,173	1,254	518	68
Q3 10.6≤ to <14.1%	1,165	1,860	1,867	1,513	71
Q4 ≥14.1%	181	622	1,221	3,752	35
**Suita High score**					
Q1 <7.4%	241	135	31	8	42
Q2 7.4≤ to <10.6%	141	228	175	104	65
Q3 10.6≤ to <14.1%	51	176	255	289	67
Q4 ≥14.1%	11	67	183	1,063	20
**Combined**					
Q1 <7.4%	4,843	1,205	393	97	26
Q2 7.4≤ to <10.6%	3,022	2,401	1,429	622	68
Q3 10.6≤ to <14.1%	1,216	2,036	2,122	1,802	70
Q4 ≥14.1%	192	689	1,404	4,815	32

CI, confidence interval; GFRcys, cystatin C-based estimated glomerular filtration rate; GRFcr, creatinine-based estimated glomerular filtration rate; IDI, integrated discrimination improvement; NRI, net reclassification improvement; Q, quartile.

^*^Initial Model: GFRcr.

^**^Updated Model: GFRcr+GFRcys.

Suita Low or Moderate score: Suita score <56.

Suita High score: Suita score ≥56.

NRI = 0.341, (95% CI, 0.305–0.377); *P* < 0.001.

IDI = 0.018, (95% CI, 0.016–0.020); *P* < 0.001.

## DISCUSSION

The present study compared the incidences of CKD based on the GFRcys and GFRcr in 29,375 TMM participants. The number of participants in the present study is the largest among previous cohort reports in the general population worldwide. The present study demonstrated that the prevalence of CKD was significantly higher when using the creatinine-based equations than when using the cystatin C-based equations. In addition, there was a positive correlation between the GFRcys and GFRcr for all participants. It has been reported that the prevalence of CKD in the general population was higher when using the creatinine-based equations than when using the cystatin C-based equations and was strongly dependent on the method used to estimate the GFR.^16^ Cystatin C is a 13-kD protease inhibitor that is produced by all nucleated cells independent of muscle mass and sex.^17^ It is well known that circulating levels of creatinine are dependent on muscle mass and aging.^18^ These observations suggest that the equation using the serum cystatin C level to estimate the GFR has smaller biases with respect to age, sex, and race compared with the equation using the serum creatinine level. Cystatin C is eliminated via glomerular filtration and is metabolized by proximal tubular cells.^17^ However, there is also extrarenal production of cystatin C.^19^ Serum levels of cystatin C reach a plateau in patients with end-stage renal disease (ESRD).^19^ In addition, cystatin C levels have been reported to be altered in patients with thyroid disorders, those receiving glucocorticoid therapy after a kidney transplant, individuals with HIV infection, and pregnant women.^20^^–^^27^ Although the present study did not measure the GFR based on the urinary clearance of iothalamate, cross-sectional analyses that included 13 studies have shown that the GFRcys is similar to the GFR based on the urinary clearance of iothalamate compared to the GFRcr and could be a useful equation to confirm CKD.^6^

Although the present study was cross-sectional and did not provide follow-up data, based on a meta-analysis of 11 studies involving the general population (*n* = 90,750) and 5 studies involving cohorts with CKD (*n* = 2,960), Shilpak et al reported that the GFRcys and the combination of the GFRcys and GFRcr are predictors of all causes of death and CVD.^28^ In addition, in a study evaluating a 10-year Framingham risk score in 468 outpatients with obesity and type 2 diabetes in Japan, Ito et al found that the GFRcys is closely correlated with CVD risk.^29^ These observations suggest that the GFRcys may be more predictive of the CVD risk than the GFRcr.

Although it is well known that hs-cTnT and NT-proBNP levels are higher in CKD patients than in patients with normal renal function,^30^^–^^33^ it is unclear whether the GFRcys or GFRcr are related to CV biomarkers in a general Japanese population. The present study has shown that the GFRcys was associated with CV biomarkers, including hs-cTnT and NT-proBNP levels, compared to the GFRcr. Some papers have shown that the cystatin C-based renal function measurement was more accurate than the creatinine-based measurement, since cystatin C is less affected by muscle mass and diet than creatinine.^34^^–^^36^ In addition, serum cystatin C may have some extrarenal mechanisms, including CV risk factors.^37^ It has been demonstrated that levels of cystatin C have been found to be associated with elevated C-reactive protein levels, suggesting that cystatin C might be involved in vascular inflammation, which then progresses to atherosclerosis.^38^^–^^40^ It has also been reported that the imbalance between cathepsins and their inhibitor cystatin C determines the progression of atheroscerosis.^41^^–^^44^ Inflammatory cytokines associated with atherosclerosis stimulate the production of lysosomal cathepsins.^41^^,^^45^^–^^47^ Cathepsins are involved in the rupture of atherosclerotic plaques.^41^^,^^45^^–^^47^

In the present study, the multiple regression models demonstrated that an increase in serum uric acid was strongly associated with decreases in both the GFRcys and GFRcr after adjusting for the baseline characteristics and UACR. Consistent with our findings, some epidemiologic studies that have evaluated the relationship between hyperuricemia and kidney disease have shown that elevated levels of uric acid are independently related to serum levels of creatinine in patients with CKD stages G1 and G2.^48^^–^^50^ The long-term follow-up of a randomized clinical trial using allopurinol or placebo found that allopurinol slowed the progression of kidney disease and reduced the onset of CVD.^51^ An epidemiologic study demonstrated that an increase in serum levels of uric acid was significantly associated with CVD events, independent of diuretic use and other CV risk factors.^52^ These observations suggest that an increase in uric acid may play an important role in the pathogenesis of CKD.

The present study also shows that increases in hs-cTnT and NT-proBNP are strongly associated with decreases in the GFRcys and uric acid. The main purpose of the present study was to find differences in renal function measurements between the GFRcys and GFRcr and determine whether the GFRcys and/or GFRcr are related to CV biomarkers and the CVD risk in the general population. Therefore, we selected NT-proBNP and hs-cTnT, which are representative CV biomarkers. The hs-cTnT is released following myocardial injury due to cardiac ischemia, for example, and possibly also cardiac strain.^53^ Similarly, NT-proBNP may be a marker of “pancardiac disease”, as it is associated with left ventricular (LV) dysfunction, LV hypertrophy, left atrial dilation, and cardiac ischemia.^54^ Mechanisms are involved in the relationship between these biomarkers and the GFR in an apparently healthy population that may have subclinical cardiac injury. Previous studies have shown that natriuretic peptide levels and cTnT levels correlate inversely with the GFR in CKD and NT-proBNP levels are influenced by renal filtering function.^30^^–^^33^ However, the pathophysiologic mechanisms causing increases in cTnT concentrations in patients with CKD are not clear.^55^^,^^56^ There is emerging evidence that increases in cTnT in asymptomatic patients with ESRD indicate subclinical myocardial damage.^55^^,^^56^ It has been suggested that elevated levels of cTnT are associated with pathologic evidence of old myocardial necrosis or microinfarction, the severity of angiographic CAD, and LV hypertrophy in patients with ESRD.^56^

The term cardio-renal syndromes (CRS) has been used to identify a disorder of the heart and kidneys whereby acute or chronic dysfunction in one organ may induce acute or chronic dysfunction in the other organ.^57^^,^^58^ It has been shown that cardio-renal interactions occur in both directions and the postulated mechanisms of the CRS progression may be attributed to the roles of low cardiac output, renal hypoperfusion, central venous congestion, neurohormonal elaboration, anemia, oxidative stress, renal sympathetic nerve activity, and other factors.^57^^,^^58^

In the Japanese medical field, the renal function measurement is usually assessed using the creatinine-based GFR and not a cystatin C-based measurement. However, it was uncertain whether either approach (creatinine vs cystatin C) is effective for predicting CVD risk. The present study has shown that the ability to predict CVD risk was improved when the GFRcys was added to the GFRcr in an assessment of the CVD risk using the Suita score.

### Study limitations

There are several limitations to the present study. First, there were no follow-up data, including mortality and the onset of CVD, because this was a cross-sectional study. Additionally, we did not examine the measured GFR using the urinary clearance of inulin. Second, all of the participants in the TMM had experienced the Great East Japan Earthquake that resulted in a tsunami and were under stress from exposure to these disasters. Therefore, we have to consider the impact of the mental or economic stress experienced by the participants in the aftermath of this disaster, although the participants in the TMM were defined as a “general” population. Although the present study investigated the comparison between the GFRcys and GFRcr to determine whether these measurements were associated with CV biomarkers and elevated CVD risk, we had to calculate the CKD stage as the parameter for the Suita score using the GFRcr.

### Conclusions

There was a significant difference in the GFR between the cystatin C- and creatinine-based equations. The GFRcys had a closer association with CV biomarkers, including hs-cTnT and NT-proBNP levels, and a high Suita score compared with the GFRcr and provides additional value in the assessment of the CVD risk using GFRcr.
